# ﻿*Aspleniumguodanum* (Aspleniaceae), a distinct new fern species from northern Guangdong, China, based on morphological data and molecular phylogeny

**DOI:** 10.3897/phytokeys.241.122789

**Published:** 2024-04-30

**Authors:** Ke-Wang Xu, Yu-Tong Han, Yu-Ran Dong, Jian-Qiang Guo, Ling-Feng Mao, Wen-Bo Liao

**Affiliations:** 1 Co-Innovation Center for Sustainable Forestry in Southern China, College of Life Science, Nanjing Forestry University, Nanjing 510275, China; 2 College of Ecology and Environment, Nanjing Forestry University, Nanjing 510275, China; 3 Administrative Commission of Danxia Mountain National Park, Shaoguan 512300, Guangdong, China; 4 State Key Laboratory of Biocontrol and Guangdong Provincial Key Laboratory of Plant Resources, School of Life Sciences, Sun Yat-sen University, Guangzhou, 510275, China

**Keywords:** Conservation, Danxia landform, molecular phylogeny, species diversity, taxonomy

## Abstract

A new spleenwort species, *Aspleniumguodanum*, was found and described from Danxia landform region in Guangdong, China. The new species has close resemblance to *A.subcrenatum* Ching ex S.H.Wu in morphology, but can be distinguished by having plants small, stipes and rachises not covered with fibrous scales, relatively fewer pairs of pinnae, pinnae short, pinna margin weakly biserrate, pinna apex acute and lower pinnae obviously reduced. Phylogenetic analyses, based on six plastid markers (*atpB*, *rbcL*, *rps*4 & *rps4-trnS* and *trnL* & *trnL-F*) of the new species and its relatives, support a close relationship between *A.guodanum* and *A.subcrenatum*. Only one population with no more than 50 individuals were found and, therefore, it is recommended to be classified as Critically Endangered (CR) following IUCN Red List Criteria.

## ﻿Introduction

*Asplenium* L. is one of two genera in the fern family Aspleniaceae, comprising more than 700 species worldwide (Xu et al. 2020). Members of the genus can be recognised by usually having erect rhizomes with radial steles, two C-shaped vascular bundles usually forming one X-shaped bundle at apex of the petiole, clathrate rhizome scales, distinctly sulcate rachises with a raised ridge in the centre and two grooves on each side, elongated sori normally located on one side of a vein (Mitsuta et al. 1980; Lin and Viane 2013; Luna et al. 2020). It has a worldwide distribution and is native to almost all parts of the world, except Antarctica and some high Arctic areas (Kramer and Viane 1990). The majority species of the genus often grow in the forest floor, on banks of rivers and in ravines in montane vegetation. Special landforms (e.g. Danxia, karst) often harbour endemic species (Kramer and Viane 1990; Schneider et al. 2005; Jiang et al. 2011; Xu et al. 2022).

China has rich landform types and is one of the diversification centres of the genus *Asplenium*. Approximately 100 species (with more than 20 endemic species) of the genus have been recorded in China and most are distributed in southern and south-western China (Chang et al. 2013, 2018; Lin and Viane 2013; Xu et al. 2018, 2019, 2022). Though extensive investigation of vascular plant has been carried out to understand the species diversity in different regions of China, some narrowly distributed species usually with extremely small populations or some cryptic species with blurred species delimitation have been overlooked.

During our field investigation of vascular plants in Danxia Mountain of northern Guangdong Province, we found a small population of a peculiar *Asplenium* species in shaded steep cliffs. Morphologically, these plants have distinctly sulcate rachises with a raised ridge in the centre and two grooves on each side, greyish-green or stramineous-green stipes and rachises, lamina 1-pinnate, falcate pinnae and serrate pinna margins. Based on these morphological characteristics, it is clearly a member of the *A.wrightii* complex (Xu et al. 2021). After comparing with species in the *A.wrightii* complex and those in the genus, we preliminarily consider it might represent a new species in the genus. Therefore, we subsequently conducted a phylogenetic analysis to confirm its species status and detect its phylogenetic position. The results support our hypothesis that it is an undescribed new species and more closely related to members of the Bullatum clade and to *A.subcrenatum*; therefore, here we describe it, based on molecular, macro- and micro- morphological evidence.

## ﻿Material and methods

### ﻿Morphological studies

All specimens studied here were deposited at the Herbaria of Nanjing Forestry University (NF) and Sun Yat-sen University (SYS). Herbarium abbreviations follow those in Index Herbariorum of website NYBG Steere Herbarium (https://sweetgum.nybg.org/science/). Observation of macromorphological characteristics were carried out both from the fresh plants and dried herbarium specimens. Rhizome scales were observed using a light microscope. Measurement of quantitative characteristics was conducted using ImageJ software (Pérez and Pascau 2013), based on digital images of voucher specimens. In order to observe the spore morphology, we use a Scanning Electron Microscope (SEM) to take spore images of the new species. Mature and fine spores were selected from the voucher specimen. We mounted them on specimen tabs and then they were coated with platinum in a sputter coater. Finally, we used an ESEM-Quanta 200 (FEI, Hillsboro, Oregon, US) with 15 Kv at Nanjing Forestry University to scan and observe the spore morphology.

### ﻿Molecular phylogenetic studies

#### ﻿Taxon sampling

To detect the phylogenetic position of the putative new species of *Asplenium*, we newly generated DNA sequence data including four plastid sequences of two separate fronds from Danxia Mountain, Guangdong Province. We also included all accessions of the *A.bullatum* clade from the previous studies of Xu et al. (2020). A total of six plastid markers of 67 accessions as the ingroup were incorporated into the molecular analyses (Suppl. material 1). The representatives of all other clades defined by Xu et al. (2020) in *Asplenium* and one representative of *Hymenasplenium* were selected as outgroups.

#### ﻿DNA extraction, PCR amplification and sequencing

Total genomic DNA was extracted from silica-gel-dried leaves using the modified 2× CTAB procedure of Doyle and Doyle (1987). Four plastid markers (the *atpB* gene, the *rbcL* gene, the *rps4* gene and the *rps4-trnS* intergenic spacer) were selected for amplification and sequencing, based on the previous phylogenetic study (Xu et al. 2020). Primers and PCR protocols followed Xu et al. (2020). Except for the new species, all other DNA sequences used in this study were downloaded from NCBI. Voucher information and GenBank accession numbers are provided in Suppl. material 1.

#### ﻿Sequence alignment and phylogenetic analysis

The newly-generated sequences were assembled and edited using Sequencher ver. 4.14 (GeneCodes Corporation, Ann Arbor, Michigan). All sequences were initially aligned with MAFFT ver. 7 (Katoh and Standley 2013) and manually adjusted in BioEdit (Hall 1999). Phylogenetic analyses of the plastid datasets were conducted using Maximum Likelihood (ML) and Bayesian Inference (BI) on the CIPRES web server (Miller et al. 2010), respectively. The ML tree searches were performed using RAxML-HPC2 on XSEDE with 1000 bootstrap replicates. The model GTR+I+G was selected for the concatenated dataset using the AIC standard of jModelTest 2 (Darriba et al. 2012). BI analysis was conducted using MrBayes 3.1.2 (Huelsenbeck and Ronquist 2001) with two independent runs of four Markov Chain Monte Carlo chains (one cold, three heated), each beginning with a random tree and sampling one tree every 1000 generations for 10,000,000 generations. Convergence amongst runs and stationarity were assessed using Tracer ver. 1.4 (Rambaut and Drummond 2007) and the burn-in was discarded. The remaining trees were used to calculate a 50% majority-rule consensus topology and posterior probabilities (PP).

## ﻿Results and discussion

### ﻿Morphological comparison

**Figure 1.**
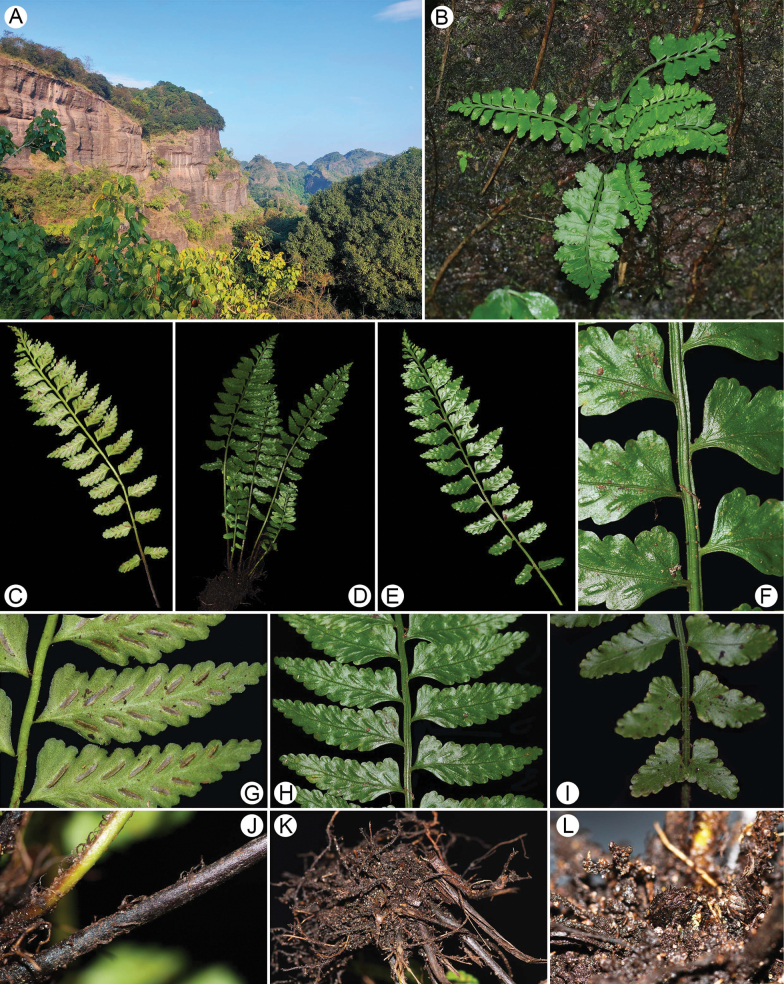
Macromorphology of *Aspleniumguodanum* sp. nov. **A** habitat **B** habit **C** abaxial lamina **D** frond **E** adaxial lamina **F** rachis **G** abaxial view of pinna **H** adaxial view of pinna **I** reduced pinna base **J** scales at base stipe **K** rhizome **L** fiddlehead.

The new species has erect rhizomes, distinctly sulcate stipes and rachises with a raised ridge in the centre and two grooves on each side, 1-pinnate laminae, often falcate pinnae and serrate pinna margins (Fig. 1). These characters support this new taxon as a member of the Aspleniumser.Wrightiana Ching & S.H.Wu recognised by Wu (1989) in his traditional classification of Chinese *Asplenium*, based on morphological data. Initially, 14 species were included in the series by Wu (1999), but most of them were treated as synonyms of *A.wrightii* Eaton ex Hook. by Lin and Viane (2013), although they noted the wide range of morphological variation of members in this series suggesting distinct, although artificial, groups. Recently, Xu et al. (2021) provided a taxonomic revision of the *A.wrightii* complex reinstating *A.alatulum* Ching and *A.subcrenatum* Ching ex S.H.Wu, based on phylogeny and morphology. Therefore, only three species (*A.alatulum*, *A.subcrenatum*, *A.wrightii*) were recognised in the *A.wrightii* complex.

**Figure 2.**
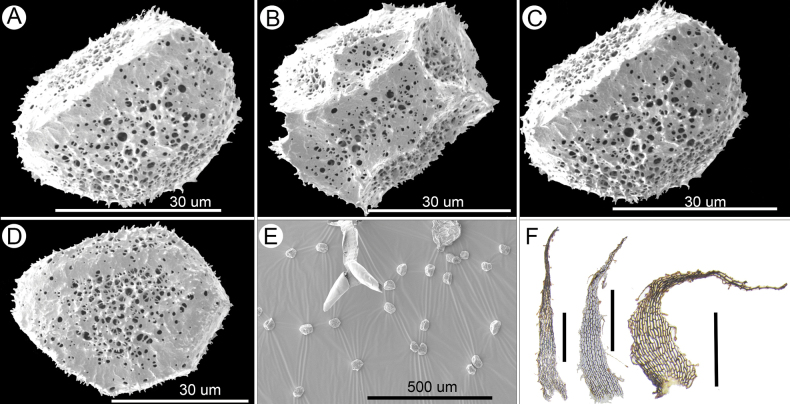
Micromorphology of *Aspleniumguodanum* sp. nov. **A–E** spore **F** scale. Scale bar: 1 mm.

The macromorphology of the putative new species is most distinct in the *A.wrightii* complex, with lower pinnae obviously reduced and pinna margin weakly biserrate (Fig. 1). Within the *A.wrightii* complex, it is most similar to *A.subcrenatum* by their crenate teeth, but can be easily distinguished from *A.subcrenatum* by the combined characters of having plants of small size, stipes and rachises not covered with fibrous scales, relatively fewer pairs of pinnae, pinnae short, pinna margins weakly biserrate, pinna apex acute and lower pinnae obviously reduced. In addition, the scales of the new species are brown to dark brown (Fig. 2), while those of *A.subcrenatum* are reddish-brown (Xu et al. 2021). The new species can be distinguished from *A.alatulum* and *A.wrightii* by having small size, few pairs of pinnae, crenate marginal teeth, lower pinnae obviously reduced and foraminate-alate perispore (Fig. 2, Xu et al. 2021).

### ﻿Molecular phylogenetic analyses

**Figure 3.**
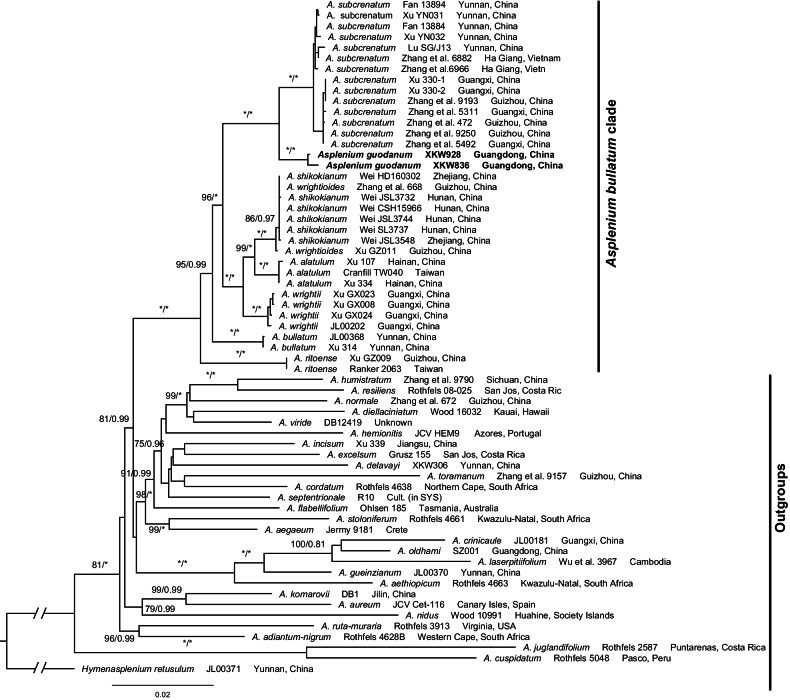
Maximum Likelihood phylogeny of the *Aspleniumbullatum* clade, based on six plastid markers (*atpB*, *rbcL*, *rps*4 & *rps4-trnS* and *trnL* & *trnL-F*). The numbers associated with branches are Maximum Likelihood bootstrap support (MLBS) and Bayesian Posterior Probability (BIPP). The asterisk indicates MLBS = 100 or BIPP = 1.00.

The alignment for phylogenetic analyses including six plastid markers was 4,748 bp, of which 3,085 sites were constant, 954 characters were parsimony informative and 709 variable characters were parsimony uninformative. A total of six sequences for the new species are newly generated for this study (Suppl. material 1). The monophyly of *Asplenium* was confirmed by our reconstructed phylogeny (Fig. 3). The putative new *Asplenium* species was strongly supported as a member of the *A.bullatum* clade (Xu et al. 2020). The sister phylogenetic relationship between the new species and *A.subcrenatum* was strongly supported (Fig. 3). In addition *A.alatulum*, *A.shikokianum* Makino, *A.wrightii* and *A.wrightioides* Christ were included in the *A.wrightii* complex and strongly-resolved sister to the subclade of the new species (Fig. 3). *Aspleniumshikokianum* was proved to be a natural hexaploid hybrid between octoploid *A.wrightii* and tetraploid *A.ritoense* Hayata (Taiwan Pteridophyte Group 2019). In our plastid phylogenetic trees, *A.shikokianum* is not nested with parental *A.wrightii*, instead nesting together with *A.wrightioides* which was also treated as a synonym of *A.wrightii* by Lin and Viane (2013). The relationship of these polyploids still needs further study using nuclear data.

### ﻿Taxonomic treatment

#### ﻿ Asplenium guodanum

Taxon classificationPlantaePolypodialesAspleniaceae

K.W.Xu & W.B.Liao sp. nov.

714B09D6-57A0-5B0E-9069-99BFB2FFE184

urn:lsid:ipni.org:names:77340982-1

##### Type.

China. Guangdong: Shaoguan City, Renhua County, Danxia Mountain, 25.013834, 113.617885, 539 m elev., 10 Nov 2023, *Jian-Quang Guo & Ke-Wang Xu 836* (holotype: SYS!; isotype: NF!).

##### Diagnosis.

*Aspleniumguodanum* is morphologically most similar to *A.subcrenatum*, but different by having small size (15–30 cm tall vs. 30–55 cm tall), rhizome scales brown to dark brown (vs. reddish-brown in *A.subcrenatum*), stipes and rachises not covered with fibrous scales (vs. densely covered with fibrous scales in *A.subcrenatum*), relatively fewer pairs of pinnae (10–15 pairs vs. 18–25 pairs), pinnae short (2–3.5 cm vs. 6–10 cm), pinna margins weakly biserrate (vs. almost entire to crenate-sinuate in *A.subcrenatum*), pinna apex acute (vs. acuminate in *A.subcrenatum*) and lower pinnae obviously reduced.

##### Description.

Plants 15–30 cm tall. Rhizomes short and erect, densely scaly; scales brown to dark brown, membranous, lanceolate, 4–4.5 × 0.5–1 mm, margins with hair-like outgrowths. Fronds caespitose; stipe dull to semi-shiny, greyish-green to brown or stramineous-green, (3–) 5–9(–13) cm, base densely scaly, scales similar to those on rhizome; lamina oblong, (8–) 10–16 (–20) × (3–)4–7 cm, base truncate, apex acute, 1-pinnate; pinnae 10–15 pairs, basal pinnae subopposite, others alternate, at an angle of 60°–80° to rachis, shortly stalked, stalks ca. 2 mm, lower pinnae obviously reduced, middle pinnae falcate-lanceolate, (1.2–)2–3.5 × 0.5–1 cm, base asymmetrical, acroscopic side truncate at an angle of 45°–60° to costa, basiscopic side cuneate, becoming decurrent on rachis in apical part of lamina, margin weakly biserrate, teeth crenate, apex acute. Veins (1 or) 2-forked, with terminal hydathode. Fronds papery, dark green abaxially and greyish-green adaxially when dry, subglabrous; rachis stramineous-green, sparsely scaly to subglabrous, scales similar to those on stipe, terete abaxially, sulcate adaxially with a raised ridge in the centre and two grooves on each side, winged towards apex. Sori linear, 2–5 mm long, usually on acroscopic veinlets, near costa; indusia greyish-brown, linear, membranous, margin entire, opening towards costa, persistent. Spores with average exospore length 50–55 μm, perispore foraminate-alate.

##### Distribution and conservation assessment.

*Aspleniumguodanum* is currently only known from Danxia Mountain, Shaoguan City, northern Guangdong. Only one population with no more than 50 individuals was found. According to IUCN Red List Criteria B2a or D (IUCN 2022), this species should be listed as critically endangered (CR). More extensive fieldwork at low elevations in nearby mountains will be needed to accurately assess its conservation status.

##### Ecology.

*Aspleniumguodanum* was observed in shaded plunging cliffs of Danxia landform, at an elevation of ca. 500 m.

##### Etymology.

The species epithet is in honour of the late professor Guo-Da Chen, based at Sun Yat-sen University for his great contributions to the Danxia landform.

##### Vernacular name.

guódá tiějiǎojué (国达铁角蕨; Chinese name).

##### Comments.

No members of the *Aspleniumwrightii* complex have been recorded in Danxia Mountain before *A.guodanum* was found (Peng 2011). This new species was found in the Danxia Mountain characterised for the Danxia landform of sandy soils. Therefore, we speculate that this species is endemic to Danxia landform. Its morphologically similar species *Aspleniumsubcrenatum* was observed to grow in karst mountains and rarely in acid soil, while *A.alatulum* and *A.wrightii* usually grow on wet, rather acid soil. Habitat heterogeneity might be the most important factor for species diversification in this species complex. In addition, it can be easily distinguished from *A.subcrenatum* by having plants of smaller size, stipes and rachises not covered with fibrous scales, relatively fewer pairs of pinnae, pinnae short, pinna margin weakly biserrate, pinna apex acute and lower pinnae obviously reduced.

### ﻿A key to *Aspleniumguodanum* and its closely-related taxa in the *A.wrightii* complex

**Table:** 

1	Pinna bipinnate	***A.shikokianum***
–	Pinna unipinnate	**2**
2	Plants up to 30 cm tall, pinnae 10–15 pairs, pinnae 2–3.5 cm in length, pinna margin weakly biserrate, lower pinnae obviously reduced	***A.guodanum***
–	Plants more than 30 cm tall, pinnae more than 10 pairs, pinnae more than 5 cm in length, pinna margin serrate, lower pinnae not or slightly reduced	**3**
3	Stipes and rachises densely scaly, scales reddish-brown, pinna margins almost entire to crenate-sinuate, mainly occurs in limestone areas	***A.subcrenatum***
–	Stipes and rachises rarely densely scaly, scales brown to dark brown, pinna margins serrate to coarsely dentate, mainly occurs in acid soil	**4**
4	Rhizomes erect to decumbent, scale cells oblong, rachises with broad lateral wings, pinnae 10–15 pairs	***A.alatulum***
–	Rhizomes erect, scale cells quadrangular, rachises only winged towards apex, pinnae (12–)17–25(–34) pairs	***A.wrightii***

## Supplementary Material

XML Treatment for Asplenium guodanum

## ﻿Additional information

### Author ORCIDs

Ke-Wang Xu https://orcid.org/0000-0003-2239-0487

Yu-Tong Han https://orcid.org/0009-0005-3916-9123

Yu-Ran Dong https://orcid.org/0000-0001-5591-1360

Jian-Qiang Guo https://orcid.org/0009-0004-2304-0360

Ling-Feng Mao https://orcid.org/0000-0002-2884-135X

Wen-Bo Liao https://orcid.org/0000-0001-6839-9511

### Data availability

All of the data that support the findings of this study are available in the main text or Supplementary Information.

## Supplementary materials

Supplementary material 1 List of voucher specimens and Genbank accession numbers used in phylogenetic analyses Data type xlsx https://binary.pensoft.net/file/1040243This dataset is made available under the Open Database License (http://opendatacommons.org/licenses/odbl/1.0/). The Open Database License (ODbL) is a license agreement intended to allow users to freely share, modify, and use this Dataset while maintaining this same freedom for others, provided that the original source and author(s) are credited.Ke-Wang Xu, Yu-Tong Han, Yu-Ran Dong, Jian-Qiang Guo, Ling-Feng Mao, Wen-Bo Liao
